# Digital health and consumer health informatics: past and future

**DOI:** 10.18103/mra.v14i1.7206

**Published:** 2026-01

**Authors:** Xia Jing, Hua Min, Yang Gong

**Affiliations:** 1 Department of Public Health Sciences, College of Behavioral, Social and Health Sciences, Clemson University, Clemson, SC, United States; 2 Department of Health Administration and Policy, College of Public Health, George Mason University, Fairfax, VA, United States; 3 Department of Clinical and Health Informatics, McWilliams School of Biomedical Informatics, The University of Texas Health Sciences Center at Houston, Houston, TX 77030, USA;

**Keywords:** digital health, consumer health informatics, perspectives, trends, challenges

## Abstract

This perspective paper introduces digital health and consumer health informatics, their focus areas, origins, current trends, and future challenges. The paper aims to elaborate on the differences and their connections within the broader context of biomedical and health informatics. The goal is to provide a better and clearer navigation system for readers and researchers in the fields of digital health and consumer health informatics.

Digital health and consumer health informatics have flourished in recent years, driven by advances in digital technology, computing power, and connected devices. Digital health and consumer health informatics both play an increasingly critical role in the general public’s health and well-being. The two fields differ in focus, origin, and mechanisms. This short paper aims to elaborate on the differences, their connections within the broader context of biomedical and health informatics, their histories, and potential future developments. Our goal is to help readers navigate these fields more efficiently by clarifying their scope, highlighting their differences, and ultimately promoting the health and well-being of the general public.

Digital health uses digital tools to enhance health and well-being and improve health outcomes. Typical digital tools include smartphones, pedometers (e.g., Fitbit), mobile apps, sensors, and other wearable devices. The focus of digital health is largely on the effectiveness and efficacy of these tools in keeping the general public or patients with certain conditions healthy and in improving patients’ condition management, medication adherence, and communication with their healthcare providers. The tools used in digital health are primarily commercial products. A 2022 report from the National Academy of Medicine used a broader definition of digital health^1^. Terms such as mhealth, ehealth, telehealth, and telemedicine are sometimes used interchangeably with digital health. Digital health typically focuses on the general public, including healthy individuals, patients, and healthcare providers.

Consumer health informatics, on the other hand, is a branch of health informatics. Consumer health informatics focuses on informatics tools and their applications among the general public. Typical informatics tools include consumer-oriented websites (e.g., MedlinePlus), personal health records, and patient portals, as well as the digital health tools mentioned earlier. The focus of consumer health informatics lies in understanding the mechanisms of these tools and how they play roles in consumers’ health and well-being. Understanding the underlying scientific foundation can illuminate the design and development of better, more effective tools to empower consumers. Consumers’ information use and the effectiveness of information during communication are the primary focuses of the field. Consumer health informatics typically focuses on the general public. Table 1 presents a side-by-side comparison of the two. Figure 1 illustrates digital health and consumer health informatics as two fields.

## The origins and the past

We examined the past research through the existing literature to identify themes and current trends in digital health and consumer health informatics.

**Figure 2:**
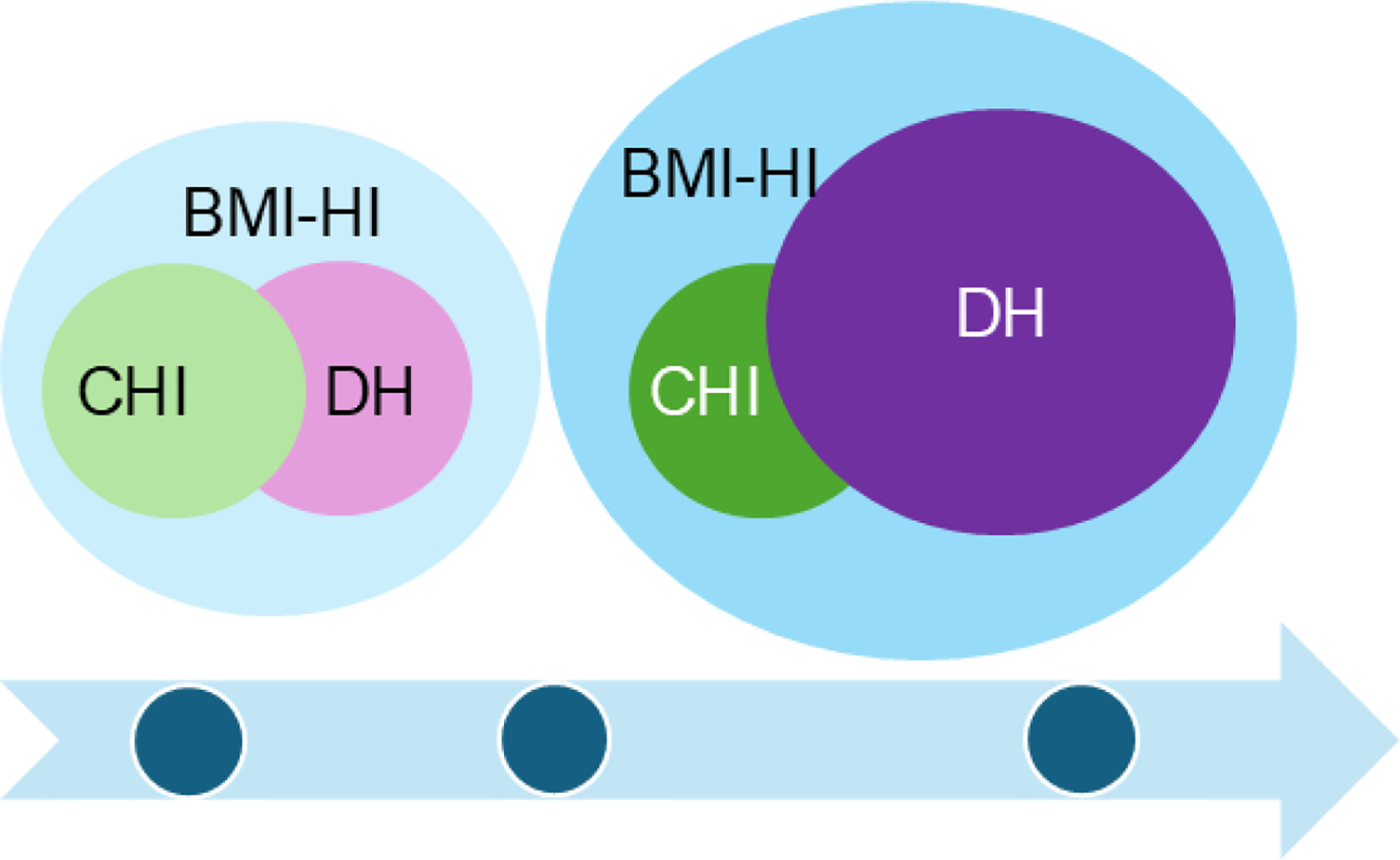
The evolution of Consumer Health Informatics (CHI) and Digital Health (DH) and their relationships to Biomedical and Health Informatics (BMI-HI)

It is hard to pinpoint the origin of the term ‘digital health’. The practice of using different-colored flags on ships to convey health-related information has been around for centuries, i.e., to communicate health information remotely. Telemedicine has been used for decades to provide needed specialty medical care remotely for civilians, the military, prison populations, and individuals living in rural areas^2,3^. Telehealth is a broader concept than telemedicine, including clinical research, education, and medical care virtually^2,3^. MeSH is a controlled vocabulary that powers PubMed, the largest biomedical and health literature database in the world. MeSH term has included digital health since 2024, which reflects a recent surge in the topic despite its long history. The definition of digital health as a MeSH term is much broader, encompassing health IT, electronic health records, telemedicine, and personalized medicine, as well as wearable devices as digital health tools. Meanwhile, it is parallel to consumer health informatics and medical informatics in MeSH. The topology shows the relationships among the three, especially the latter two, which were more maturely defined and have been included in MeSH for much longer (Figure 2). In addition, the starting years of several digital health journals also reflect the field’s trajectory to some extent. Digital Health (SAGE journal) started in 2015, the Lancet Digital Health in 2019, and BMC Digital Health in 2023.

Digital health projects utilize commercial products directly, evaluating their effectiveness and efficacy. Digital health interventions typically use text messages, phone calls^4^, mobile apps, or alerts through electronic health records or patients’ portals. The scopes of the studies are quite broad including but not limited the following areas, demonstrated through either systematic reviews or randomized control trials: chronic conditions management^5^, cancer screening^4^, behavioral changes^6,7^, eating disorders^8^, mental health^9–11^, fall prevention and detection^12^, medication management and healthcare service delivery^13^, Parkinson’s disease^14^, attention deficit hyperactivity disorder (ADHD)^15^, pediatric oncology^16^, musculoskeletal conditions^17^, stroke^18^, heart failure^19^, multiple sclerosis^20^, overweight and obesity^21,22^, suicide prevention^23^, antibiotic prescription^24^, hypertension management^25^, chronic kidney disease^26^, as well as well-being promotion and maintenance, such as sleep, step counts, screentime monitoring, etc. Many of the digital health studies are collaborations between academia and industry. The targeted populations range from children, adolescents^6^, to seniors^5,7,12,27^. Some studies focus on urban settings, and others focus on rural settings^27^. Many studies focused on individuals with chronic conditions, while others focused on healthy adults^7^. Digital health tools can improve older adults’ physical activities with good evidence^7,28^. However, the digital tools’ true effects on outcomes for individuals with overweight and obesity are mixed despite clear evidence of better engagement via digital tools, which suggests digital tools alone may not be adequate to achieve the ideal health outcomes in these cases^21,29^. The geographic areas also have good coverage, including North America, Africa^13^, and Asia countries^25,30^.

1993 might be the starting point of the term “consumer health informatics”^31^. However, the papers relevant to consumer health informatics can be traced back to 1965. The early papers focus more on consumer health or consumer health information rather than on formal consumer health informatics, as a subject^31^. The names reflect the focuses of the early efforts in consumer health informatics and the transition overtime, very information-focused and consumer-focused. Consumer health informatics research spans from patient education and communication to health education and involves health record keeping via patient portals or personal health records. Typically, how to provide information more effectively is a primary focus of the field. Demiris’s 2016 paper provided an excellent summary of consumer health informatics progress over the prior 25 years, although it did not distinguish between digital health and consumer health informatics at the time. The first theme discussed in the paper is home telehealth^31^, and mHealth (mobile health) was listed as a theme under the consumer health informatics field. This indicates the perceived relationship between the two at the time. Consumer health informatics has been included as a MeSH term since 2018, and it is positioned parallel to digital health. Another relevant MeSH term is consumer health information, which was introduced into MeSH in 2008.

In consumer health informatics, the studies fall into the following focuses: precision prevention, particularly in chronic condition prevention^32^, facilitating communication of medical texts and reports to patients^33^, social media and online platforms to detect and manage disease outbreaks^34^, the role of visualization in chronic condition management^35^, prostate cancer prevention among black men^36^, e-consent among patients for sharing their health information^37^, online information source to facilitate patient reported measures among stroke patients^38^, and mobile apps utilized among pregnant women^39^. Self-developed tools, such as personal health records, can be used in consumer health informatics research. Researchers aim to improve these tools by identifying their underlying mechanisms. Although the ultimate goal is to improve the health and well-being of the general public, the pathways to achieving this goal can differ from those in digital health. Figure 2 shows the evolution of consumer health informatics and digital health over time and their relationships to the broader context of biomedical and health informatics.

## The future

In this section, we share our perspectives on digital health and consumer health informatics, outlining possible directions, current challenges, and research and development trajectories over the next several decades.

Artificial intelligence (AI) has reemerged with the impressive performance of large language models (LLMs) and has attracted significant attention across scientific and everyday life. Inevitably, AI will be a key technology applied, refined, and further developed in both digital health and consumer health informatics. However, the roles of AI in these two fields may differ in nuanced ways despite their shared overarching goal of improving human health and well-being. In digital health, the future of AI applications may still heavily focus on assisting individuals in promoting and maintaining their health through both software and devices. On the other hand, in consumer health informatics, the focus of the AI technology may align with the primary target population: the general public, to obtain health information via AI assistants or its elaboration, explanation, and alternative representation via AI generation.

In both digital health and consumer health informatics, more and more automated tracking data can support data-driven optimization across various applications. By analyzing aggregated tracking data, the content and the display could be adjusted to better serve the general public and provide more precise solutions. In both fields, AI will play a more significant role as a collaborator, better serving the general public more effectively and precisely. Other future directions may be heavily influenced by the opportunities outlined in the next section, given the current challenges in both fields.

## Challenges and opportunities

The primary challenges in digital health can be exemplified in at least three areas: device and service costs, additional demands on already-strained healthcare providers’ workloads, and the need to provide authentic health information in this “generative era”. Most digital health interventions involve devices, commercial apps, or newly developed sensors, some of which can be costly. This could further worsen the health disparity among different socioeconomic groups. In addition, many digital health interventions, especially those targeted at particular conditions, such as hypertension^25,40–43^ or type 2 diabetes^42,44^, require healthcare providers’ constant feedback or timely actions to achieve ideal outcomes. In theory, this might be an ideal practice for patients and their healthcare providers to maintain good communication and make timely adjustments to treatment plans based on patients’ real-time condition, ultimately improving patients’ health outcomes. In practice, this practice not only could worsen healthcare providers’ burnout and make their workload even more unmanageable, but without corresponding reimbursement reform to recognize healthcare providers’ contributions outside of office or hospital encounters, this demand, despite its purely good intentions, could also be unrealistic to sustain. Meanwhile, if the reimbursement is made without a well-planned, robust, and well-thought-out plan, this could lead to another fraud hotspot.

The effectiveness of digital health tools still needs rigorous evaluation. One meta-analysis showed that such tools are effective in improving physical activities among older adults; however, other medical-related measures, such as depression or hospital days, did not show statistically significant results^5^. In addition, the paper also recommends expanding the functionality of digital tools to include more healthcare-related services^5^. This indicates that digital interventions need more comprehensive functionality beyond the basics. Another recommendation is greater implementation at individual homes, in addition to community-level implementation of digital technologies, and some are in healthcare provider settings^27^. Regarding the data generated and reported by patients or the general public, how to use such data by both the general public and providers, and how to maximize its benefits, is not a simple question that can be easily answered^45^.

The challenges for consumer health informatics primarily lie in the following aspects: consumer digital literacy has improved significantly over the last few decades, especially among younger generations, who are fairly tech-savvy, which raises requirements for user engagement and information utility while providing the corresponding services. Another huge challenge is the needed workforce to fight back against the constantly generated false health information mixed with true information. Since the LLM can generate fluent languages in scientific or academic contexts, this does not imply the authenticity or validity of the information. The information generated certainly does not ensure that the sources of information are scientifically valid and explicitly documented. Discerning legitimate from false health information is a critical skill for everyone; unfortunately, not everyone can do so. Placing the burden of educating the general public and fighting back against false health information solely on healthcare providers is another unrealistic and unsustainable expectation on already-strained healthcare providers. A dedicated workforce with the right background knowledge, training, and understanding of health and medicine is needed at an accelerated pace, given the rapid pace of generated health information and its dissemination. Another challenge for healthcare providers is keeping up with increasingly demanding consumers. For example, through a PHR or patient portal, patients may be able to identify discrepancies in their records and request updates from the healthcare provider. Considering the ratio between patients and healthcare providers, the requests, even if they are not from every single patient for every single encounter, could be significant to meet.

Another challenge across both digital health and consumer health informatics is that high-quality, rigorously designed and conducted randomized controlled trials (RCTs) are not yet a common practice in either field^7,10^, which affects the robustness of the evidence generated and the confidence in disseminating the results on a large scale. In addition, a longer follow-up is recommended to obtain robust evidence^9,28^, and more personalized interventions^9^, precise solutions^46^, and tailored solutions^47^ are also recommended. Long-term engagement of end users in digital health has been identified as a primary challenge for digital health interventions to truly impact the outcomes. In addition, a scale study^10^ and further analysis on cost and effectiveness are also needed^4^. Broader adoption of digital tools is still needed in African countries^13,18^. The strategies need to go beyond information or technology accessibility^47^. User engagement is a first step that has been proven to help both digital health apps and consumer health informatics information sources; however, the true effect of the intervention is much more complicated and challenging to achieve^39^. Another interesting finding is the identification of needs for providers and caregivers’ support for multiple sclerosis patients^20^. In addition, it seems that advanced natural language processing (NLP) techniques have not been broadly adopted to facilitate the communication of medical texts to patients^33^. Limited progress on racial and ethnic minority groups in consumer health informatics^48^. Although patient education on prostate cancer focuses on black men, it is a rare but excellent example^36^.

A further challenge involves the vast amount of data generated by personal devices and how these data can be integrated with medical records seamlessly. Ensuring interoperability between consumer-generated data and clinical systems, maintaining data standards, accuracy, and consistency, and protecting patient privacy are critical issues that must be addressed to make these data useful for clinical decision-making and research. Future opportunities include leveraging advanced analytics and AI to extract actionable insights and creating seamless, secure integration between personal devices and clinical systems to support personalized care.

In summary, digital health and consumer health informatics share the same target population, the general public. The differences primarily lie in the tools used and the study goals in each field, although there are significant overlaps between the two. Although we think the distinction between the two is nuanced, better and more precise definitions for them can help practitioners better identify their work, using accurate keywords to describe their work, better align their work with the work in the same field, better educate future work force in the fields, and ultimately to greatly improve human health and well-being via digital tools and informatics tools.

## Figures and Tables

**Figure 1:**
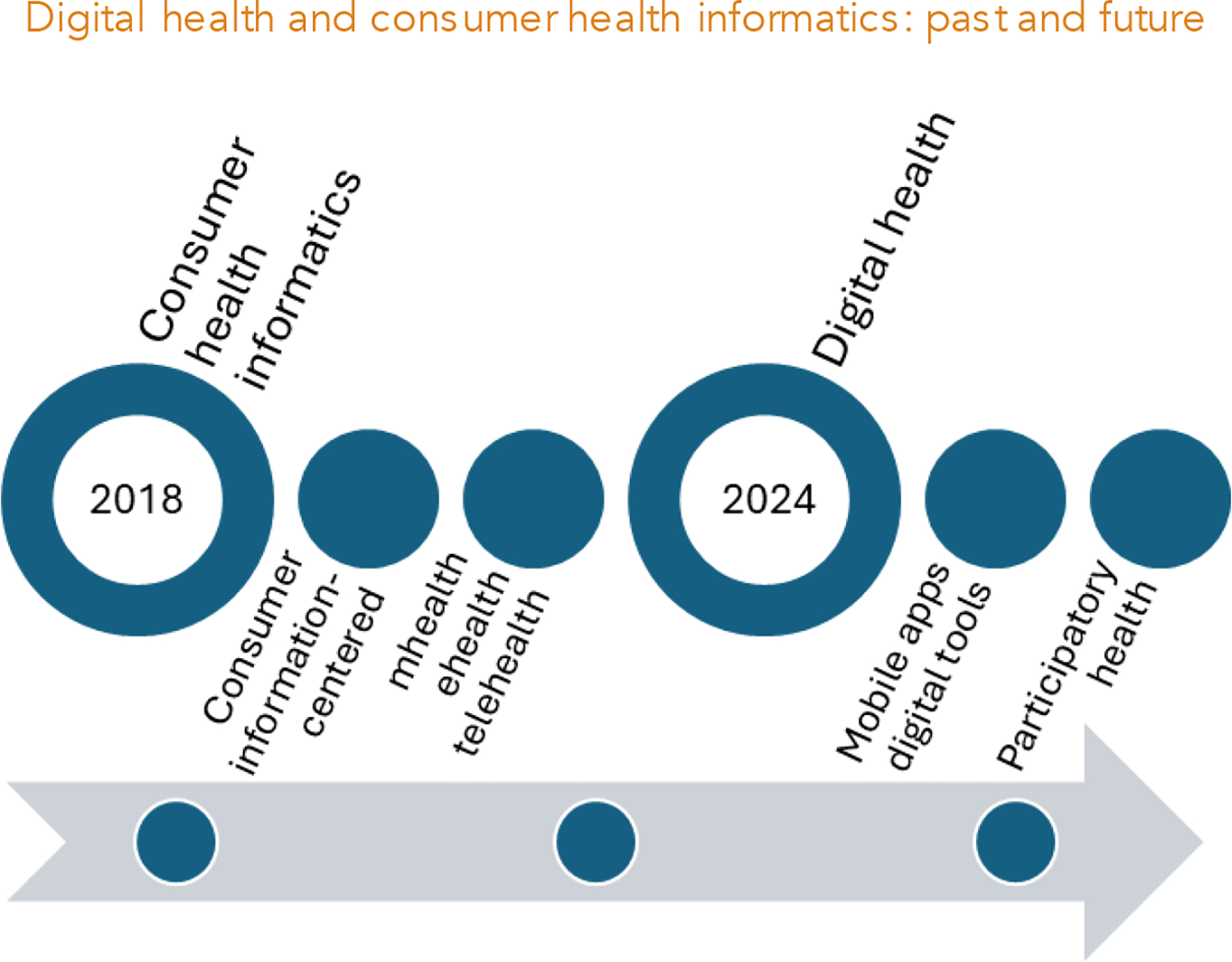
Consumer Health Informatics and Digital Health

**Table 1:** Comparison of digital health and consumer health informatics

	Digital health	Consumer health informatics
**Goals**	Effectiveness or efficacy evaluation of commercial products on health and well-being programs	Information usage and deciphering the underlying mechanism to improve
**Hardware**	Advanced medical, nonmedical, personal-use devices	Personal-use devices
**MeSH definition**	Use of digital technologies in medicine and other health professions to manage illnesses and health risks and to promote wellness	The field devoted in informatics from multiple consumer or patient views
**MeSH term start year**	2024	2018
**Software**	Programs, app, api, algorithms used in digital tools, e.g., electronic health record (EHR), clinical decision support system (CDSS), telemedicine platforms, digital therapeutics, AI/machine learning algorithms, etc	Programs, app, api used in consumer-facing tools, e.g., patient portal, personal health record (PHR), wellness apps, symptom checkers, diet and fitness apps
**Study objects**	Commercial products	Commercial products or self-developed technology/prototypes
**Target population**	General public + Individuals seek healthcare + healthcare providers	Patient population or the general public
